# Symptomatic Exercise-induced Intraventricular Gradient in Competitive Athlete

**DOI:** 10.5935/abc.20170075

**Published:** 2017-07

**Authors:** Helder Dores, Lígia Mendes, António Ferreira, Jose Ferreira Santos

**Affiliations:** 1 Hospital da Luz Setúbal, Setúbal - Portugal; 2 Hospital da Luz Lisboa, Lisboa - Portugal; 3 Hospital das Forças Armadas, Lisboa - Portugal; 4 NOVA Medical School, Lisboa - Portugal

**Keywords:** Athletes, Echocardiography, Stress, Heart Ventricles/physiopathology, Exercise Test/adverse effects, Ventricular Dysfunction/etiology

## Case Report

We describe the case of a 17-year-old caucasian male tennis player, training a mean of 20-24h/week, refereed for evaluation in Sport’s Cardiology clinic due to symptoms of dizziness on strenuous exercise, relieving soon after decubitus. The athlete denied other concomitant complaints, namely thoracic pain, palpitations, syncope or decrease in physical performance. Although this is the most symptomatic episode, he revealed other prior episodes with similar presentation, but less intense and occurring in environments with high temperatures. Personal/family history was unremarkable and all pre-competitive evaluations were normal and without restrictions for competitive sport. Physical examination did not show significant findings - cardiac evaluation was normal, heart rate and blood pressure at rest were 52 bpm 121/64mmHg respectively.

**Figure 1:**
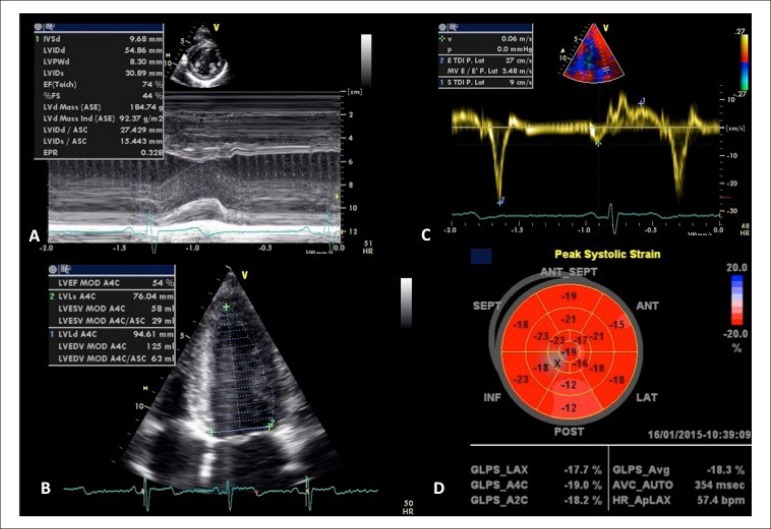
Transthoracic echocardiogram at rest without evidence of significant morfo-functional abnormalities - left ventricle dimensions (LV) by M Mode (A), volumes and LV ejection fraction (B), tissular Doppler at mitral ring (C) and global longitudinal strain (D).

**Figure 2:**
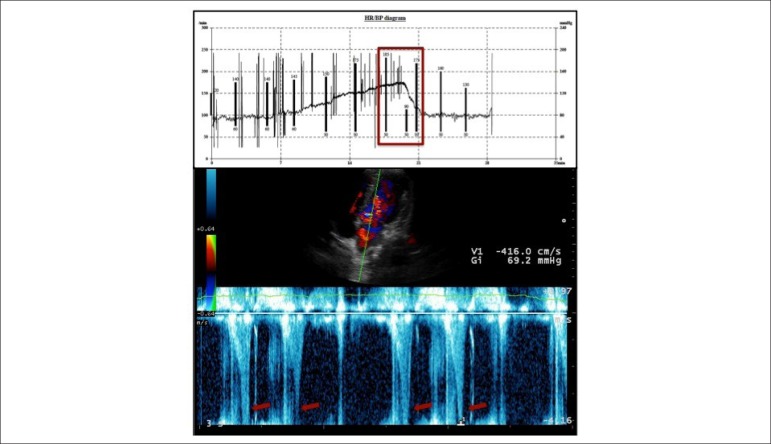
Exercise stress echocardiogram performed in treadmill with Bruce protocol, revealing a significant decrease in systolic blood pressure (185 ➔ 90mmHg) in peak of exercise, with concomitant detection of IVG (bottom picture).

The 12-lead electrocardiogram and transthoracic echocardiogram did not show pathological findings, only cardiac physiological adaptations to exercise (Figure 1). Subsequently the athlete underwent a treadmill exercise stress echocardiogram revealing an excellent functional capacity (19’09’’ of Bruce protocol, 19.3METs), but with reproduction of symptoms (dizziness) in the peak of exercise with simultaneous decrease in systolic blood pressure (185➔90mmHg) and detection of intraventricular gradient (IVG) - at least 69mmHg (Figure 2). In the first minute of recovery the symptoms disappeared and blood pressure normalized.

The athlete was advertised to stop the sportive practice. An ambulatory 24h-Holter monitoring and cardiac magnetic resonance were subsequently performed, not showing pathological changes, namely arrhythmias or structural cardiac abnormalities.

After these investigations the case was discussed with involvement of the athlete, parents and coach. It was decided to reinitiate exercise with a gradual increase in intensity and volume of training, with the special advertising to increase hydration (apparently suboptimal according to the coach report) and to begin beta-blocker therapy if the symptoms persist. After 18 months of follow-up the athlete remain asymptomatic, with excellent performance and without need of pharmacologic therapy.

## Discussion

The development of significant exercise-induced IVG (>30mmHg at rest or >50mmHg with exercise) is uncommon, but can lead to several and unspecific symptoms such as dizziness, thoracic pain, or even ventricular repolarization changes and arrhythmias during exercise test.^1,2^ This condition is usually associated to global or segmental left ventricular hypertrophy or an abnormal implantation of the papillary muscles, but the pathophysiological mechanisms are not well established. Three potential mechanisms are purposed for the development of IVG:

a) Increase of physiological non-obstructive IVGs;
b) End-systolic obstruction secondary to ventricular cavity obliteration;
c) Mid-systolic obstruction due to systolic anterior motion of the mitral valve with restriction of ejection flow.^3,4^

In a study performed by Zywca et al.^5^ the independent predictors of dynamic left ventricular outflow tract obstruction in individuals without hypertrophic cardiomyopathy were: chordal systolic anterior motion, smaller left ventricle at end-systole, higher systolic blood pressure at peak, younger individuals and increased septal wall thickness.^5^ However, as in the case reported, IVG can occur without structural cardiac changes, namely of the mitral valve apparatus, and eventually justified by extreme myocardial deformation in response to load conditions.^3^ In this context, IVG is more frequently described in athletes or in situations with increased inotropic stimuli as during dobutamine stress echocardiogram.^6,7^ Exercise stress echocardiogram plays a relevant role in the evaluation of symptomatic athletes, with reproduction of symptoms and the potential detection of significant IVGs.^1,8^

The clinical significance of IVG remains unknown - it could be one extreme physiological adaptation to exercise, one isolated pathological entity or in the other hand corresponds to a pre-phenotypic finding of cardiomyopathy.

Regarding the preventive/therapeutic measures to adopt in the presence of an athlete with IVG, maintenance adequate hydration during exercise is crucial, often sufficient for the remission of symptoms. Exercising under higher temperatures without adequate hydration can increase the gradient secondary to left ventricle cavity obliteration. Among the pharmacological therapy, the evidence indicates a significant effectiveness of beta-blocker therapy, both in the remission of symptoms and in the remission/disappearance of IVG.^1,9^

The small published data and the short follow-up of athletes with IVG did not permit definite conclusions regarding the prognostic impact, but there are not described fatal clinical events in athletes with IVG without structural cardiac changes. In this setting there are not specific recommendations relatively to competitive sport in athletes with IVG.^10,11^ In general, if an athlete is still symptomatic despite the stressed preventive/therapeutic measures, it is not advised to maintain sportive practice, especially with the intensity of exercise that precipitates the symptoms, and this should be regularly evaluated during follow-up.

Shortly, in the presence of an athlete with exercise-induced symptoms, IVG should be taken in consideration. The exclusion of potential pathologies associated to an increased risk for sudden cardiac death is fundamental in the reproduction of symptoms, in which exercise stress echocardiogram plays an important role. IVG remains poorly clarified and some questions unanswered:

- Which is the etiology/pathophysiology of IVG (physiologic versus pathologic)?
- Which is the clinical impact at long-term of IVG?
- Which should be the recommendations regarding the eligibility for competitive sport of athletes with IVG?
- Which should be the surveillance/follow-up of athletes with IVG?
